# The Enhance-Fuse-Align Principle: A New Architectural Blueprint for Robust Object Detection, with Application to X-Ray Security

**DOI:** 10.3390/s25216603

**Published:** 2025-10-27

**Authors:** Yuduo Lin, Yanfeng Lin, Heng Wu, Ming Wu

**Affiliations:** 1 Guangdong Provincial Key Laboratory of Cyber-Physical System, School of Automation, Guangdong University of Technology, Guangzhou 510006, China; 2 College of Engineering, Shantou University, Shantou 515063, China; 3 School of Computer, Guangdong University of Technology, Guangzhou 510006, China; 4 Department of Computer Science, KU Leuven, 3000 Leuven, Belgium; 5 Department of Mechanical Engineering, KU Leuven, 3000 Leuven, Belgium

**Keywords:** object detection, signal degradation, security screening, multi-scale feature fusion, enhance-fuse-align principle

## Abstract

Object detection in challenging imaging domains like security screening, medical analysis, and satellite imaging is often hindered by signal degradation (e.g., noise, blur) and spatial ambiguity (e.g., occlusion, extreme scale variation). We argue that many standard architectures fail by fusing multi-scale features prematurely, which amplifies noise. This paper introduces the Enhance-Fuse-Align (E-F-A) principle: a new architectural blueprint positing that robust feature enhancement and explicit spatial alignment are necessary preconditions for effective feature fusion. We implement this blueprint in a model named SecureDet, which instantiates each stage: (1) an RFCBAMConv module for feature Enhancement; (2) a BiFPN for weighted Fusion; (3) ECFA and ASFA modules for contextual and spatial Alignment. To validate the E-F-A blueprint, we apply SecureDet to the highly challenging task of X-ray contraband detection. Extensive experiments and ablation studies demonstrate that the mandated E-F-A sequence is critical to performance, significantly outperforming both the baseline and incomplete or improperly ordered architectures. In practice, enhancement is applied prior to fusion to attenuate noise and blur that would otherwise be amplified by cross-scale aggregation, and final alignment corrects mis-registrations to avoid sampling extraneous signals from occluding materials.

## 1. Introduction

Object detection in complex imaging environments represents one of the most persistent challenges in computer vision, where traditional approaches frequently fail due to inherent domain-specific limitations. Across diverse fields, from medical diagnosis to security screening and remote sensing, practitioners encounter fundamental obstacles including noise interference, multiscale object variations, occlusion handling, and feature degradation that significantly compromise detection accuracy and reliability. Conventional object detection frameworks, originally designed for natural image scenarios characterized by clean backgrounds, consistent lighting, and well-defined object boundaries, are often ill-equipped for real-world applications. These applications require robust performance in environments that can be demonstrably affected by severe noise, atmospheric interference, overlapping structures, and variable acquisition conditions. For example, in medical imaging, radiologists face the critical task of detecting subtle tumors within noisy CT scans, where inherent contrast variations and imaging artifacts can obscure vital diagnostic features [1]. Security personnel face the daunting task of identifying concealed contraband in heavily cluttered X-ray baggage imagery with extensive object occlusion [2]; see also advances in real-world X-ray benchmarks and detection models [3,4,5]. In addition, enhanced detonator detection in X-ray baggage via image manipulation and deep CNNs has been explored [6], and robust perception under adverse visibility has leveraged thermal imaging for human and vehicle detection [7,8]. Beyond security screening, real-time object detection for assistive navigation further exemplifies the need for efficient detectors [9]. Remote sensing analysts struggle with small object detection in vast-scale variations while contending with atmospheric effects and complex terrain heterogeneity [10,11]. While recent deep learning advancements have individually demonstrated considerable potential to address specific challenges for multi-domain object detection, a significant impediment to developing truly robust systems is the absence of a unified framework that systematically integrates feature enhancement [12], data fusion, and spatial alignment. Current methodologies are often siloed, typically excelling in one area at the expense of others. For example, some methods prioritize noise reduction without effectively leveraging multiscale features [13,14], while others focus on fusion while neglecting the critical issue of spatial misalignment inherent in oriented object detection [15].

This paper introduces the Enhance-Fuse-Align (E-F-A) principle, a comprehensive framework that systematically addresses the three fundamental pillars of robust object detection in challenging domains. The E-F-A principle encompasses (1) Enhancement of degraded features through adaptive attention mechanisms, (2) Fusion of multiscale contextual information using bidirectional feature pyramids, and (3) Alignment of spatial representations to handle orientation and scale variations. We demonstrate this principle through SecureDet, a novel architecture specifically designed for X-ray contraband detection that achieves state-of-the-art performance while maintaining computational efficiency suitable for real-time security screening applications.

Foundational detectors and architectural primitives have laid the groundwork for modern object detection: YOLOv3 popularized single-stage real-time detection with multi-scale predictions, Feature Pyramid Networks (FPN) introduced top-down multi-scale feature fusion, and deformable convolutions enabled adaptive spatial sampling for geometric variations, which our E–F–A design builds upon and extends in challenging X-ray settings [16,17,18]. For security X-ray imaging, large-scale benchmarks such as SIXray and the GDXray NDT dataset have been instrumental for evaluation and algorithm development [19,20].

Consequently, the central contribution of this work is not solely the introduction of the SecureDet detector, but rather the proposal and rigorous validation of a general architectural blueprint: the Enhance-Fuse-Align (E-F-A) principle. We contend that for domains like X-ray security screening, which are marked by severe signal degradation and spatial ambiguity, the specific ordered sequence of operations—first enhancing features, then performing spatial alignment, and finally fusing multi-scale representations—is of paramount importance. SecureDet serves as our instantiation designed to rigorously test this principle. To ensure a fair evaluation that isolates the benefits of E-F-A, we utilize YOLOv8 as the baseline backbone. Extensive experimentation, including critical ablation studies that disentangle module roles and their ordering, unequivocally demonstrates that this principled, disciplined design yields superior results compared to simply assembling advanced components, thereby clearly distinguishing our contribution from routine YOLO variants.

## 2. Related Work

### 2.1. Challenges in Medical Imaging Object Detection

Medical imaging presents unique challenges where noise, artifacts, and anatomical complexity significantly impact object detection performance. Tumor detection in noisy CT scans exemplifies these fundamental challenges, where low-dose imaging protocols introduce substantial noise while preserving diagnostic accuracy remains critical. Zhang et al. [21] demonstrated how annotation quality directly impacts segmentation performance in mandible segmentation of CT images, revealing that even small amounts of label noise can severely degrade the effectiveness of the deep learning model. Karimi et al. [22] provided a comprehensive analysis of noise-resistant training methods, identifying data scarcity and annotation quality as the main bottlenecks in medical image analysis. New methods are also being developed to recover high-quality images from degraded sources by using diffusion models [23] to guide the restoration process [24]. Recent advances in noise reduction for medical imaging have focused on preserving diagnostic features while minimizing artifacts. Mileto et al. [13] evaluated iterative reconstruction algorithms combined with CNN-based denoising, showing that while traditional iterative methods modify spatial resolution non-linearly, deep learning approaches can maintain diagnostic quality. Al-Antari et al. [25] introduced the Quadratic CNN (Q-CNN) architecture that enhances sensitivity to analyze noisy radiographs without requiring training on noisy images, demonstrating superior robustness compared to conventional approaches. These medical imaging challenges parallel those found in security screening, where object detection must operate reliably despite image degradation and complex backgrounds.

### 2.2. Remote Sensing and Satellite Imaging Challenges

Remote sensing represents another domain where fundamental imaging challenges mirror those in security applications. Yang et al. [10] identified critical issues in oriented object detection including feature misalignment, spatial misalignment, and periodicity of angle problems that cause training instability. The challenge of small object detection across vast scale variations is particularly acute in satellite imagery, where objects occupy few pixels while maintaining detection accuracy across diverse geographical contexts [26]. Wei et al. [27] addressed multi-scale attention for small object detection in remote sensing, highlighting insufficient utilization of small object information and weak robustness against complex backgrounds. These challenges directly parallel X-ray security screening scenarios where small contraband items must be detected within cluttered baggage environments. Liu et al. [28] provided comprehensive analysis of small object detection in aerial images, emphasizing inadequate positive samples and inaccurate localization—problems that extend across multiple challenging imaging domains. Dubovik et al. [11] outlined grand challenges in satellite remote sensing, identifying atmospheric effects and sensor limitations that create fundamental trade-offs between spatial coverage and resolution. These atmospheric and environmental degradation effects mirror the image quality issues encountered in medical CT imaging and X-ray security screening, establishing common technical foundations across diverse application domains.

At the architectural level, widely used primitives include channel–spatial attention modules (CBAM) and efficient channel attention (ECA) for feature enhancement, spatial pyramid pooling (SPP) for multi-scale context aggregation, and bidirectional feature pyramid fusion as instantiated in EfficientDet for cross-scale aggregation, which we build upon in our E–F–A design [29,30,31,32]. In addition, multi-resolution post-filtering with attention for image quality enhancement and cross-modal infrared–visible image fusion further motivate our enhancement and fusion strategies [33,34]. Within the architectural primitives, FPN provides a top-down pathway with lateral connections for multi-scale feature fusion, while BiFPN extends this idea with bidirectional cross-scale connections and learnable per-edge fusion weights for iterative refinement, which we adopt as the fusion operator in SecureDet [32]. For completeness, classic baselines such as Faster R-CNN and SSD established, respectively, the two-stage RPN-based pipeline and the single-stage dense default-box formulation that underlie many subsequent detectors [35,36].

## 3. Method

### 3.1. Overall Architecture and Design Philosophy

**Figure 1:**
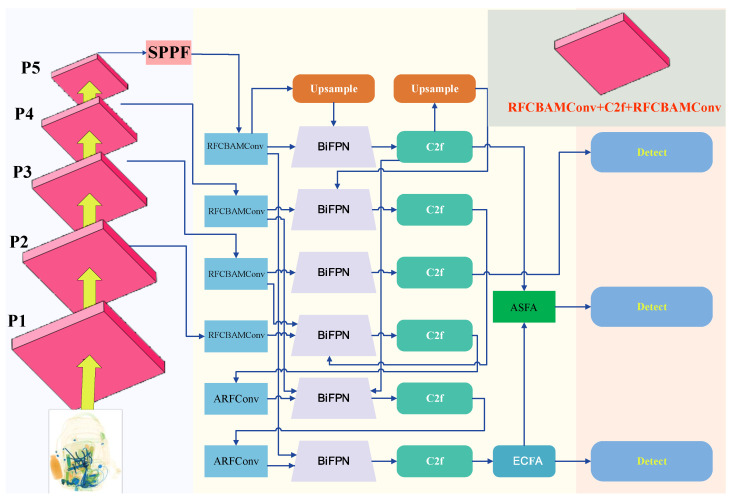
The overall architecture of the SecureDet model. The SecureDet architecture is engineered for robust multi-scale feature fusion. It employs a Bidirectional Feature Pyramid Network (BiFPN) as its foundational backbone, which is synergistically augmented with an Asymmetric Connection, an Adaptive Spatial Feature Fusion (ASFA) module, and an Edge-aware Context Feature Aggregation (ECFA) module. Specifically, the ECFA module addresses contextual alignment, while the ASFA module is dedicated to spatial and geometric alignment. This integrated framework culminates in the generation of multi-level feature maps—P3, P4, and P5 (with corresponding strides of 8, 16, and 32)—thereby enabling precise detection of objects across a wide range of scales.

The architecture of SecureDet is predicated on the Enhance-Fuse-Align (E-F-A) principle. As illustrated in Figure 1, the workflow begins with a backbone network that extracts multi-scale features from a 3-channel (RGB) input image at a 640 × 640 resolution. By integrating RFCBAMConv and C2f+RFCBAMConv modules, the backbone yields four feature maps: P2 (160 × 160, 128 channels), P3 (80 × 80, 256 channels), P4 (40 × 40, 512 channels), and P5 (20 × 20, 1024 channels). Among these, the high-resolution P2 map is critical for capturing the fine-grained details required for small object detection.

The core of the architecture is the “Fuse” stage, orchestrated by a BiFPN. The efficacy of BiFPN stems from its use of learnable weights for feature fusion, which allows the network to prioritize more informative features across different scales. This is realized through a bidirectional data flow: a top-down pathway propagates high-level semantic context, while a bottom-up pathway conveys precise spatial localization information. Within this fusion framework, we introduce a key “Enhance” strategy focused on the P2 feature map. Specifically, the P2 map (160 × 160, 128 channels) first undergoes a convolutional operation to match the dimensions of P3 (80 × 80, 256 channels). This transformed P2 map is then integrated with both the original backbone P3 feature and the P3 feature from the top-down pathway. This tripartite fusion injects the detailed spatial information from P2 into the P3 level, substantially improving localization accuracy. The entire bidirectional fusion process is applied iteratively to ensure a comprehensive synthesis of semantic and spatial features, ultimately producing P3, P4, and P5 feature maps unified at a 256-channel dimension.

Following fusion, the architecture proceeds to the “Align” stage. The fused P3, P4, and P5 feature maps are further refined by the Enhanced Context Fusion Attention (ECFA) and Attentional Self-Feature Augmentation (ASFA) modules. Finally, these refined multi-scale feature maps are fed into the detection head to perform the final object detection task.

### 3.2. Enhance Module—RFCBAMConv

**Design Rationale:** While standard attention modules like CBAM are effective, their reliance on a fixed receptive field limits performance, particularly on X-ray images characterized by blurred boundaries and large-scale variations. To overcome this limitation, we propose the Receptive Field Context-aware CBAM (RFCBAMConv). Unlike conventional approaches, RFCBAMConv first establishes a rich, multi-scale spatial context before the channel and spatial attention mechanisms are applied. This preemptive enrichment of features enables the attention module to operate on inputs that already encode diverse spatial patterns. Consequently, this design enhances the model’s ability to focus on discriminative information under challenging conditions, while simultaneously attenuating noise and improving the signal-to-noise ratio of features for subsequent fusion.

The RFCBAMConv is designed to enhance feature representation through the integration of multi-scale spatial context with adaptive channel and spatial attention mechanisms. The processing pipeline is structured as follows:

Initially, a channel attention mechanism, adopting principles from the Squeeze-and-Excitation (SE) block, is employed to generate channel weights $M_{\mathrm{ch}}\in R^{B\times C\times 1\times 1}$. Input features $X\in R^{B\times C\times H\times W}$ are first passed through global average pooling to derive per-channel statistics as shown in Equation (1):

(1) $$z_{c}=\frac{1}{H\times W}\sum_{i=1}^{H}\sum_{j=1}^{W}X_{c}\left(i,j\right)$$

Statistics are subsequently processed within a bottleneck structure that consists of two fully connected layers featuring a reduction ratio r=16 and a sigmoid activation function.

Concurrently, multi-scale receptive field features are generated. A k×k depth-wise separable convolution is utilized to capture local spatial patterns, followed by a spatial unfolding operation that integrates diverse contextual information into an enriched feature map $F_{\mathrm{unfold}}$. Subsequently, a spatial attention mechanism computes saliency maps $M_{\mathrm{sp}}$ over $F_{\mathrm{unfold}}$ through the concatenation of max and average pooling statistics across channel dimensions, followed by the passage through a 3×3 convolutional layer with sigmoid activation, as described in Equation (2):

(2) $$M_{\mathrm{sp}}=\sigma \left({\mathrm{Conv}}_{3\times 3}\left(\left[\max_{c}\left(F_{\mathrm{unfold}}\right),{\mathrm{avg}}_{c}\left(F_{\mathrm{unfold}}\right)\right]\right)\right)$$

Finally, dual-attention modulation is applied. The unfolded features $F_{\mathrm{unfold}}$ are sequentially weighted by $M_{\mathrm{ch}}$ ($F_{\mathrm{ch}}=F_{\mathrm{unfold}}⊙M_{\mathrm{ch}}$) and subsequently by $M_{\mathrm{sp}}$ ($F_{\mathrm{att}}=F_{\mathrm{ch}}⊙M_{\mathrm{sp}}$). These attention-refined features are fed into the output layer of the module: a k×k convolution with stride *k* for spatial downsampling and channel transformation, followed by Batch Normalization and ReLU activation, resulting in the final output Y as formulated in Equation (3):

(3) $$Y=\mathrm{ReLU}(\mathrm{BN}\left({\mathrm{Conv}}_{k\times k,\;s=k}\left(F_{\mathrm{unfold}}⊙M_{\mathrm{ch}}⊙M_{\mathrm{sp}}\right)\right))$$

**Figure 2:**
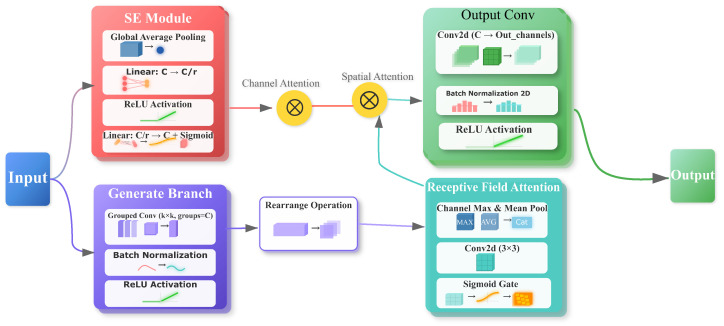
Receptive Field CBAM Convolution (RFCBAMConv) Module Architecture. Sigmoid activations are confined to attention maps (channel and spatial) for weight estimation; the main feature path applies Batch Normalization and ReLU, avoiding saturation and maintaining stable gradients.

This integrated, hierarchical design allows RFCBAMConv to first identify globally important feature types (channels) and then dynamically pinpoint their precise spatial locations using learned local descriptors. By leveraging rich multi-scale context, the module effectively focuses on discriminative features, demonstrating superior capability in handling complex imagery, such as the overlapping and semi-transparent characteristics of X-ray images, as illustrated in Figure 2.

**Implementation logic and parameter selection.** RFCBAMConv first builds multi-scale spatial context via spatial unfolding on local descriptors (depth-wise separable k×k), then applies dual attention sequentially—channel attention with a bottleneck (reduction r=16, sigmoid) followed by spatial attention obtained from concatenated max/avg channel statistics passed through a 3×3 convolution with sigmoid; finally, an output ${\mathrm{Conv}}_{k\times k,\;s=k}$ performs spatial downsampling and channel transformation, followed by Batch Normalization and ReLU. This “enhance-then-attend” ordering places attention on context-enriched features, improving robustness under noise and blur.

As shown in Table 1, the RFCBAMConv component hyperparameters provide detailed configuration settings for optimal performance.

### 3.3. Fuse Module—BiFPN

The BiFPN module processes the enhanced feature pyramid $P_{3}^{\prime },P_{4}^{\prime },P_{5}^{\prime }$ generated by the Enhance module. Its central innovation is the conceptualization of each bidirectional pathway (top-down and bottom-up) as a distinct feature network layer. These layers can be stacked iteratively to facilitate progressively higher-level feature fusion. The fusion operation itself employs a fast normalized weighted sum. Specifically, for a node combining multiple input features $I_{i}$, the output feature *O* is computed as:

(4) $$O=\mathrm{Conv}\left(\sum_{i}\frac{w_{i}}{\sum_{j}w_{j}+\epsilon }\cdot I_{i}\right)$$

Here, $w_{i}$ denotes a learnable weight for each input feature $I_{i}$. These weights are constrained to be non-negative via a post-weight ReLU activation, explicitly enabling the network to learn the relative importance of each input feature map. The constant ϵ (typically $10^{-4}$) is added to the denominator to guarantee numerical stability. Subsequently, the aggregated weighted sum is passed through a depthwise separable convolution, followed by Batch Normalization and activation functions, yielding the final fused feature map. Within the SecureDet architecture, BiFPN functions as the principal F-module, iteratively performing weighted fusion across multiple scales. Its repeated bidirectional cross-scale connections and weighted feature aggregation yield a feature pyramid rich in both high-level semantic context and fine-grained spatial details. This comprehensive representation forms a robust foundation for the subsequent alignment and detection stages.

### 3.4. Align Module

#### 3.4.1. Enhanced Contextual Feature Alignment (ECFA)

**Design Rationale:** Effectively resolving severe occlusions requires that a model perceives both global scene context and fine-grained local details. Conventional feature pyramids, however, often sacrifice the latter for the former, creating a fundamental trade-off that compromises localization precision. To address this challenge, we designed the ECFA module. It synergistically combines a Pyramid Spatial Pooling (PSP) backbone to capture long-range context with a Tanh-gated local attention mechanism to preserve and refine spatial boundaries. This dual-path architecture ensures the integration of global context while maintaining the high-fidelity local information critical for distinguishing overlapping objects. Consequently, the resulting contextual alignment suppresses spurious responses induced by clutter and stabilizes features against degradation, thereby significantly enhancing localization robustness.

**Figure 3:**
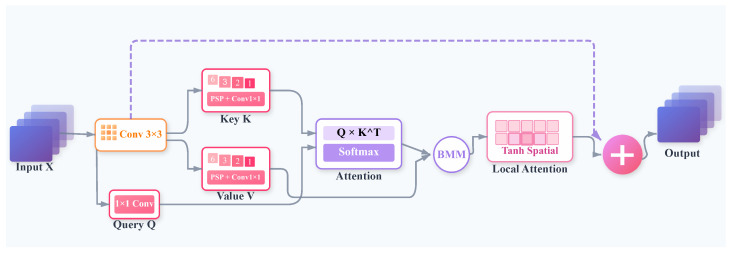
Enhanced Contextual Feature Alignment (ECFA) Module Architecture.

The enhancement of neural network feature representations is facilitated by the ECFA module through the adaptive integration of multi-scale contextual information, alongside the refinement of spatial characteristics by means of attention mechanisms, as illustrated in Figure 3. Both long-range contextual dependencies and fine-grained local spatial relationships are captured, thus improving feature discriminability for complex visual perception tasks.

The module begins with initial receptive field expansion and channel attenuation. Input features $F\in R^{C\times H\times W}$ undergo a 3×3 convolution to reduce channel dimension, yielding $F^{\prime }={\mathrm{Conv}}_{3\times 3}\left(F\right)\in R^{C/2\times H\times W}$. This stabilizes feature representations and prepares them for subsequent multi-scale processing. Pyramid Spatial Pooling (PSP) then aggregates context from diverse spatial scales by applying adaptive average pooling across predefined grid sizes (e.g., 6×6, 3×3, 2×2, 1×1) to $F^{\prime }$, generating multi-scale feature maps for the cross-scale attention stage.

During the cross-scale attention stage, spatially preserved features $F^{\prime }$ are transformed into Queries (Q), while multi-scale pooled features are mapped to Keys (K) and Values (V) via 1×1 convolutions. The interaction between Q and K is regarded as essential for the learning of relevant contextual dependencies. Attention weights A are computed using Equation (5):

(5) $$A=\mathrm{Softmax}\left(Q\cdot K\right)\in R^{HW\times S}$$

These weights drive the weighted aggregation of Value vectors, effectively distilling multi-scale context into a refined feature map C. Here, *S* denotes the number of pooled scales used in PSP. A subsequent local refinement module further processes these features by generating a spatial attention mask M using a bottleneck convolutional pathway (1×1 conv → 3×3 conv) and a Tanh activation. Contextual features C are then element-wise modulated by M and fused with the original C via a residual connection, yielding an enhanced feature map $C^{\prime }$.

The ECFA module’s final output Y is produced via a direct residual connection, integrating refined contextual features $C^{\prime }$ with the initial channel-reduced features $F^{\prime }$ as shown in Equation (6):

(6) $$Y=F^{\prime }+C^{\prime }\in R^{C/2\times H\times W}$$

Original input information is preserved while dynamically aggregated and spatially calibrated context is leveraged, enhancing feature discriminability.

#### 3.4.2. Adaptive Spatial Feature Alignment (ASFA)

**Figure 4:**
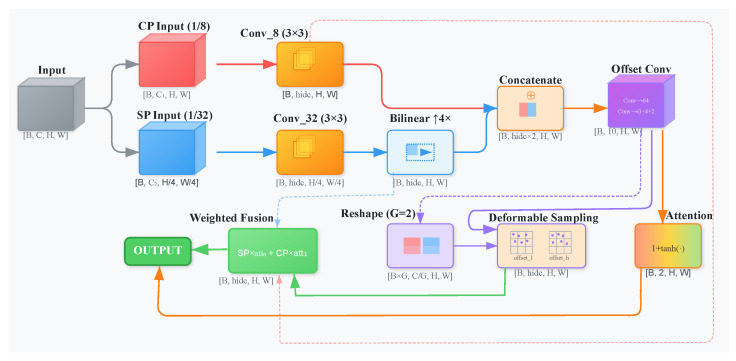
Adaptive Spatial Feature Alignment (ASFA) Module Architecture. The semantic branch applies a 3×3 convolution followed by 4× bilinear upsampling before fusion.

The ASFA module is designed to enhance multi-scale feature fusion by enabling adaptive spatial alignment and calibration through learnable spatial transformations and attention mechanisms. As shown in Figure 4, the Adaptive Spatial Feature Alignment module architecture demonstrates the dual-stream processing approach for contextual and semantic features. This module employs a dual-stream architecture, processing contextual features ($X_{\mathrm{cp}}$) and semantic features ($X_{\mathrm{sp}}$) separately. Prior to fusion, low-resolution semantic features undergo a 3×3 convolutional transformation followed by bilinear upsampling to match the spatial resolution of contextual features, as formulated in Equation (7):

(7) $$F_{\mathrm{sp}}=I\left({\mathrm{Conv}}_{3\times 3}^{32}\left(X_{\mathrm{sp}}\right)\right)\in R^{N\times C_{\mathrm{hid}}\times H\times W}$$

High-resolution contextual features are extracted concurrently via an independent 3×3 convolutional layer. The deformable sampling mechanism represents the core innovation of ASFA. Preprocessed features are concatenated and input into an offset prediction network, which generates spatial offsets $\left(\Delta_{l},\Delta_{h}\right)$ and attention weights (A). Subsequently, these learnable offsets are utilized to deform a standard sampling grid, with adaptive sampling locations computed as illustrated in Equation (8):

(8) $$G_{l}\left(i,j\right)=G_{\mathrm{base}}\left(i,j\right)+\frac{\Delta_{l}\left(i,j\right)}{{\left[W,H\right]}^{T}}$$

The adaptive spatial transformation is employed to achieve precise realignment of features, thereby enhancing robustness to geometric variations. It also reduces the sampling of noisy or occluding pixels by steering the deformable grid toward informative target structures, thereby decreasing false positives under degraded X-ray signals. The spatially calibrated features and contextual features are subsequently integrated through an attention-weighted mechanism, resulting in the production of the final output as articulated in Equation (9):

(9) $$Y=W_{1}⊙{\tilde{F}}_{\mathrm{sp}}+W_{2}⊙{\tilde{F}}_{\mathrm{cp}}$$

where $W_{1}$ and $W_{2}$ are defined as learned attention weights. The ‘Groups’ strategy (G = 2) has been incorporated to balance representational capacity with computational efficiency, thereby rendering ASFA effective for tasks that necessitate fine-grained multi-scale feature integration.

## 4. Experiments and Results

### 4.1. Experimental Configuration and Protocol

**Figure 5:**
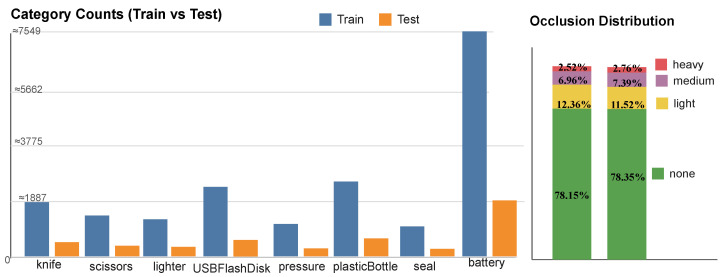
Dataset overview and statistics: training set contains 7780 images and 18,885 instances; test set contains 1944 images and 4671 instances; total is 9724 images and 23,556 instances. Eight categories are included: knife, scissors, lighter, USBFlashDisk, pressure, plasticBottleWithaNozzle, seal, battery. Occlusion distribution (by instances): training—none 78.15%, light 12.36%, medium 6.96%, heavy 2.52%; test—none 78.35%, light 11.52%, medium 7.38%, heavy 2.76%; total—none 78.27%, light 12.19%, medium 7.05%, heavy 2.57%. Occlusion levels are defined by the proportion of object area occluded: none (0%), light (<20%), medium (20–50%), heavy (>50%).

Experiments were conducted on an NVIDIA RTX 4090 workstation using Python/PyTorch with Ultralytics YOLOv8. As shown in Figure 5, comprehensive dataset statistics demonstrate the diversity and complexity of the evaluation benchmarks. The training protocol for X-ray contraband detection uses a private dataset with strong augmentation and regularization to enhance robustness and generalization. Cross-domain evaluation on OPIXray and HiXray is included. Key settings: 640×640 input resolution, batch size of 16, and 300 epochs; losses combine BCE for classification/objectness with CIoU for bounding-box regression; optimization employs SGD (initial learning rate 0.01) with a cosine schedule and warmup, EMA, weight decay, and light label smoothing. Inference uses confidence and NMS IoU thresholds of 0.25 and 0.7, respectively. Performance is reported using Precision, Recall, mAP@0.5, and mAP@0.5:0.95, together with Params, GFLOPs, and FPS. Early stopping is based on validation mAP@0.5:0.95, and the cross-domain evaluation substantiates generalization. Architectural scaling parameters of SecureDet variants (n/s/m) are summarized in Table 2 for quick reference.

As shown in Table 3, the baseline training and inference configurations provide standardized parameters for fair comparison across all evaluated models.

### 4.2. Comprehensive Performance Analysis and Benchmarking

This work performed extensive benchmarking of SecureDet against 13 state-of-the-art detection models, spanning anchor-based (Fast R-CNN [37]), transformer-based (RT-DETR [38]), efficiency-optimized (EfficientDet), and recent YOLO variants (v8–v13 [39,40,41,42,43,44]). Table 4 summarizes comprehensive performance metrics: precision (P), recall (R), mAP@0.5, and mAP@0.5:0.95, alongside critical computational efficiency indicators such as parameters, GFLOPs, and FPS. SecureDet demonstrates consistent superiority across all model scales; its flagship SecureDet-m variant achieves exceptional performance (92.60% P, 74.61% R, 82.26% mAP@0.5, 72.31% mAP@0.5:0.95), substantially outperforming the YOLOv8s baseline (+2.97% mAP@0.5, +5.93% mAP@0.5:0.95) and even surpassing the stronger YOLOv11m by +1.58% recall and +0.62% mAP@0.5:0.95, while maintaining real-time inference. Architectural comparisons reveal SecureDet’s advantages: against RT-DETR-R50, it yields +7.91% higher mAP@0.5 and +10.88% higher mAP@0.5:0.95 with 15.8% fewer parameters (19.2 M vs. 22.8 M) and comparable inference speed (+0.9% FPS; 59.2 vs. 58.7); against EfficientDet-d2, it achieves improved accuracy at comparable computational cost. Furthermore, SecureDet consistently delivers favorable accuracy-efficiency trade-offs across its model scales; the lightweight SecureDet-n variant outperforms YOLOv8n with reduced parameter count, and SecureDet-m offers an optimal balance of high accuracy, manageable parameters, and efficient computation. These results are obtained under consistent evaluation configurations, demonstrating both individual module contributions and their combinatorial effects. Complementing its broad performance gains, SecureDet exhibits outstanding localization precision, as demonstrably proven by its robustness across varying Intersection over Union (IoU) thresholds. The substantial improvements in mAP@0.5:0.95 (+5.93% over YOLOv8s, +0.62% over YOLOv11m) directly signify enhanced spatial accuracy that persists even under stricter localization criteria. This inherent robustness confirms that SecureDet not only increases the detection rate for contraband items but also localizes them with superior spatial fidelity compared to existing methods. Such high localization precision is critically imperative for automated security screening systems, where accurate object boundary delineation is fundamental for effective threat assessment and minimizing false positives or negatives.

### 4.3. Ablation Study

To systematically evaluate the independent and synergistic influences of each proposed module, comprehensive ablation experiments were conducted on the private dataset, with detailed quantitative results presented in Table 5 across eight distinct configurations. The analysis commenced by establishing the YOLOv8s baseline performance at 79.29% mAP@0.5. Individual module evaluations revealed contrasting effects: the BiFPN module, when applied in isolation, caused a marked performance degradation to 77.93% mAP@0.5 (−1.36%), indicating susceptibility to noise amplification in the absence of robust feature processing. In contrast, the RFCBAMConv module alone yielded a positive impact, improving performance to 79.85% mAP@0.5 (+0.56%), thus underscoring the inherent value of enhanced feature extraction via adaptive receptive fields. Subsequent examinations of module combinations illuminated critical architectural dependencies and limitations. The ECFA+ASFA pairing synergistically boosted performance to 80.79% mAP@0.5 (+1.50%), confirming complementary roles. However, integrating BiFPN with this combination (ECFA+ASFA+BiFPN) reduced performance to 77.88% mAP@0.5, and the RFC+BiFPN configuration achieved only 78.95% mAP@0.5, both reinforcing the reliance of BiFPN on high-quality, contextually enriched input features. Furthermore, the RFC+ECFA+ASFA configuration, despite its increased parameter count, underperformed the simpler ECFA+ASFA pair (78.82% vs. 80.79% mAP@0.5), highlighting that advanced backbone features were suboptimally utilized without an effective fusion mechanism, thereby hindering information flow. Together, these results corroborate that performing enhancement before fusion mitigates degradations (noise/blur) and prevents their amplification across the pyramid, while the final alignment stage further limits extraneous sampling from occlusions. In striking contrast to partial configurations, the full SecureDet-s model, synergistically integrating all four modules, achieved peak performance metrics: 81.63% mAP@0.5 and 69.54% mAP@0.5:0.95. These figures represent substantial absolute improvements of +2.34% and +3.16%, respectively, over the baseline. Critically, this holistic architecture demonstrated remarkable parameter efficiency, employing merely 7.55M parameters—a 45% reduction compared to the more complex RFC+ECFA+ASFA configuration (13.22 M)—while delivering superior accuracy. These empirical findings yield three overarching insights: first, the order and integration strategy of modules are more pivotal than individual capabilities; BiFPN, for instance, transforms from detrimental to significantly beneficial contingent upon receiving properly processed input features. Second, a clear hierarchical dependency governs model efficacy: RFCBAMConv lays the foundation with robust feature extraction, ECFA+ASFA refine these features through contextual enhancement and spatial alignment, and BiFPN optimally fuses this high-quality, processed information. Third, the complete architecture’s superior accuracy and efficiency validate the holistic design approach, confirming its suitability and efficacy for advanced X-ray contraband detection.

### 4.4. Dataset Evaluation

To further validate the generalization capability of our approach, we evaluated SecureDet on two public X-ray security screening datasets: OPIXray and HiXray. The results, summarized in Table 6, demonstrate the superior performance of our method compared to several state-of-the-art YOLO models. Additionally, for our private X-ray dataset, we employed a 70/20/10 split for training/validation/testing, strictly preventing sample leakage between partitions; for the public datasets (OPIXray, HiXray), we followed the official splits provided by the dataset maintainers.

On the OPIXray dataset, SecureDet achieved a mAP@0.5 of 91.16%, outperforming the next best method, YOLOv9s, which scored 90.49%. More importantly, our model achieved a recall of 87.11%, a significant improvement over other models. This high recall is critical in security applications, as it minimizes the risk of missing dangerous items.

On the HiXray dataset, SecureDet also demonstrated excellent performance. It achieved a recall of 81.11% and a mAP@0.5 of 82.59%, surpassing other YOLO variants. These results confirm the effectiveness and generalization capability of our proposed method across different datasets and contraband categories. The consistent improvements in both precision and recall validate that SecureDet provides a more reliable solution for real-world security inspection systems.

Our findings demonstrate the effectiveness and generalization of the proposed approach across diverse datasets and contraband categories. Notably, our model exhibits performance variations between the OPIXray (mAP@0.5: 91.16%) and HiXray (mAP@0.5: 82.59%) datasets. This discrepancy primarily arises from the distinct foundational challenges inherent to each dataset. OPIXray, specifically constructed to address contraband detection under severe occlusion (e.g., knives), centers on mitigating spatial ambiguity. Our SecureDet architecture, particularly its alignment modules (ECFA and ASFA), is engineered to tackle precisely these spatial misalignments and contextual confusions, leading to its superior performance on this benchmark. Conversely, HiXray, derived from authentic airport security scans, incorporates a wider array of items (e.g., power banks, liquids, cosmetics). This diversity introduces pronounced material ambiguity (where different organic substances can exhibit similar X-ray signatures) and significant intra-class morphological variations. While HiXray also contains occlusions, its primary challenges are more pronounced in discerning material properties and morphological characteristics than solely spatial ambiguities. Consequently, even though our model maintains strong performance exceeding baselines on HiXray, a degree of performance variation is understandable and expected, given its architectural emphasis on spatial occlusion resolution when confronted with these more complex, multifaceted challenges.

The experimental results demonstrated the effectiveness of the method proposed in this paper for security item detection tasks. An enhancement in the model’s capability for detecting safety threat items was observed, accompanied by a high recognition rate. This advancement provides a new technical path for the improvement of the reliability of security inspection systems.

### 4.5. Visual Performance Analysis

Comprehensive insights into SecureDet’s performance across diverse challenging scenarios are presented through a detailed visual analysis of detection results on representative X-ray security screening cases. This qualitative evaluation is complemented by quantitative metrics, illustrating the practical effectiveness of architectural innovations in real-world conditions.

#### 4.5.1. Ablation Study Visualization Analysis

**Figure 6:**
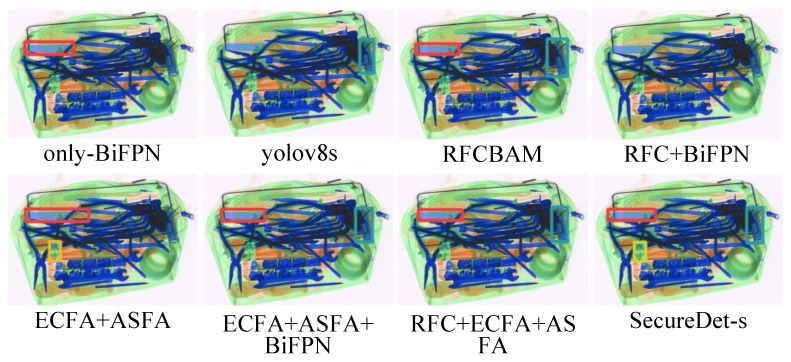
Ablation study visualization results showing SecureDet’s superior detection performance across different model configurations. The figure demonstrates comprehensive evaluation of eight different architectural combinations on challenging X-ray luggage images with dense, overlapping electronics. SecureDet-s achieves perfect precision and recall while baseline methods struggle with missed detections of small and occluded objects. Detailed quantitative metrics are provided in Table A1. “GT” denotes “Ground Truth”.

Figure 6 illustrates the detection performance of eight module configurations on a challenging X-ray luggage image, characterized by dense, overlapping, and varied-density electronics, which is representative of scenarios encountered in X-ray security screening. Moderate detection performance was exhibited by the baseline YOLOv8s, while the detection of small and occluded objects was often missed. The performance of BiFPN in isolation was noted to be inadequate, resulting in fewer detections and imprecise localization, thereby confirming its negative impact. Enhancements in feature discrimination and object boundary definition were attributed to RFCBAMConv, while improvements in spatial precision for small and boundary items were facilitated by ECFA + ASFA.

Combinations were characterized by mixed results. RFC+BiFPN yielded limited multi-scale gains; however, false positives and imprecision were introduced. ECFA+ASFA+BiFPN regressed, with confusion observed in overlapping regions, thereby highlighting the input dependence of BiFPN. RFCBAM+ECFA+ASFA exhibited inconsistencies in the presence of clutter, with a lack of effective fusion noted. The complete SecureDet-s model achieved comprehensive detection capabilities, accurate localization of objects across varying scales, occlusions, and congestion. This observation confirms the synergistic integration of all four modules, which contributed to substantially enhanced visual detection.

#### 4.5.2. Severe Occlusion Scenario Analysis

**Figure 7:**
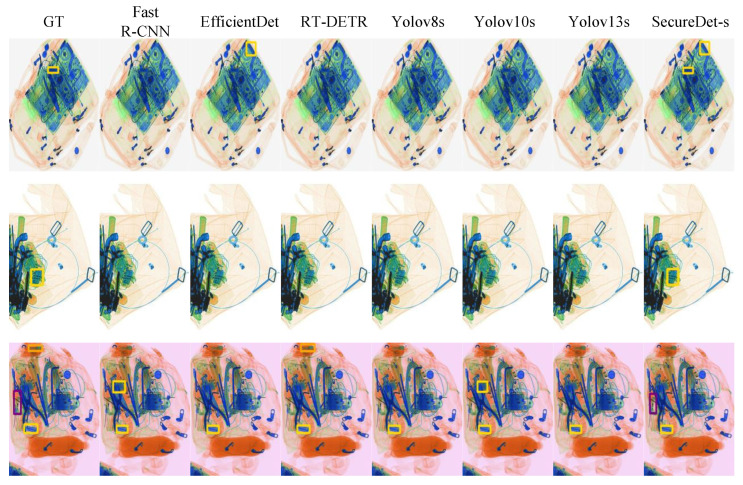
Severe occlusion scenario analysis demonstrating SecureDet’s superior performance in detecting contraband items under heavy occlusion conditions. SecureDet-s achieves the highest detection accuracy across all scenarios. Detailed quantitative metrics are provided in Table A1. “GT” denotes “Ground Truth”.

Figure 7 presents a visual evaluation of the detection performance of eight models across three X-ray baggage scanning scenarios, progressively designed to assess robustness under increasing object complexity and occlusion levels. The first scenario, characterized by moderate object overlap and semi-transparency typical of X-ray imaging, involved the identification of a single primary contraband item. Under conditions of partial occlusion, Fast R-CNN, YOLOv8s, YOLOv10s, and YOLOv13s failed entirely to detect the target, while EfficientDet and RT-DETR exhibited minor localization deviations, which were directly attributable to the occluded nature of the object. In sharp contrast, SecureDet-s achieved precise target localization with bounding boxes highly consistent with ground truth, demonstrating the capability to effectively address this level of occlusion. As the scenarios progressed to the second stage, featuring denser object stacking and greater occlusion, detection difficulty significantly amplified. Here, SecureDet-s emerged as the sole model capable of accurately detecting the contraband, maintaining high fidelity with ground truth bounding boxes, thereby confirming superior performance under more severe occlusive challenges. The third and most complex scenario involved numerous objects and extreme occlusion, which severely degraded the localization accuracy of most models—Fast R-CNN, EfficientDet, and YOLOv10s produced false detections, and YOLOv13s missed two items. Despite these extreme conditions, SecureDet-s displayed remarkable robustness, with predicted bounding boxes remaining highly proximate to ground truth, notwithstanding one missed detection and minor localization differences. This resilience is crucial, as conventional methods often struggle to identify local outliers within clusters of varying densities, a problem analogous to detecting a small, occluded item in a cluttered bag [45]. Collectively, these results highlight the exceptional capability of SecureDet-s in occlusion analysis, demonstrating enhanced robustness for complex X-ray baggage scanning tasks where overcoming occlusion is paramount.

#### 4.5.3. Small Detection Performance

**Figure 8:**
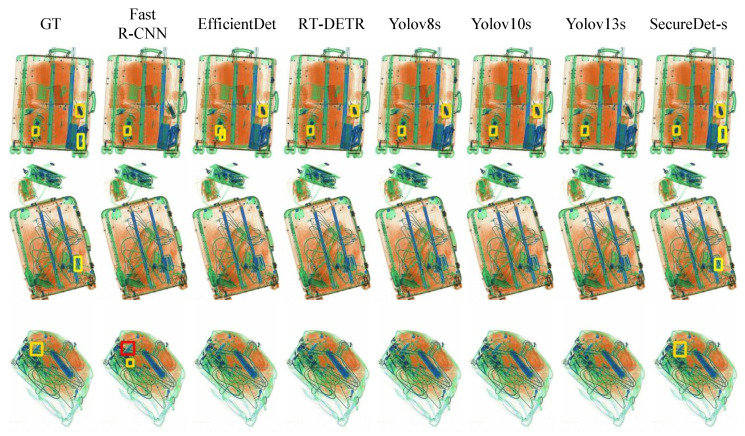
Small detection performance analysis showing SecureDet’s superior capability in detecting small objects in X-ray security screening. SecureDet-s achieves perfect detection while other methods fail. Detailed quantitative metrics are provided in Table A1. “GT” denotes “Ground Truth”. Red boxes indicate predicted bounding boxes for knives by baseline detectors; when no corresponding GT exists, they represent false positives. Specifically, in the second series scenario, Fast R-CNN’s red box marks a knife prediction where none is present, i.e., a misdetection.

SecureDet-s demonstrates excellent, consistent, and robust performance in small object detection within challenging X-ray security imaging environments, characterized by complex overlapping structures, low-contrast features, and sparse small objects. As illustrated in Figure 8 (middle row), the unique detection and precise localization of a minute object with subtle features in a demanding test case was achieved by SecureDet-s. This capability starkly contrasts with leading state-of-the-art detectors—including Fast R-CNN, EfficientDet, RT-DETR, YOLOv8s, YOLOv10s, and YOLOv13s—all of which failed to identify this critical target. The advanced ability of SecureDet-s to perceive and accurately locate tiny, indistinct objects is highlighted by this outcome. Across the entire test set, remarkable detection consistency is maintained by SecureDet-s, with precise detections closely aligning with Ground Truth (GT) annotations (as exemplified in Figure 7, first row) and reliable performance on difficult samples where frequent missed detections are exhibited by other models. When compared to other advanced methods, distinct advantages are offered by SecureDet-s: superior stability and higher recall rates are achieved over the YOLO series (v8s/v10s/v13s) for challenging small object detection tasks. In the context of X-ray imagery’s characteristic low-contrast and complex backgrounds, enhanced feature extraction and scene adaptability is exhibited by SecureDet-s compared to EfficientDet and RT-DETR. Robust detection consistency is provided by SecureDet-s, surpassing that of Fast R-CNN. The proven ability of SecureDet-s to deliver stable and reliable detection in these demanding environments underscores its significant potential for applications requiring exceptionally high precision in small object detection, such as security screening, threat detection, and medical imaging diagnostics.

#### 4.5.4. Multi-Scale Detection Performance

**Figure 9:**
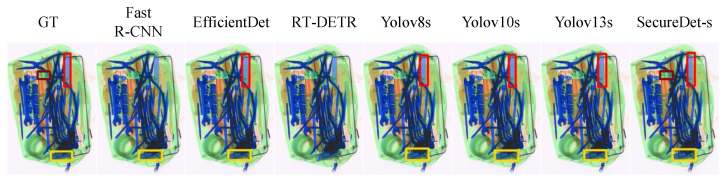
Multi-scale detection performance analysis demonstrating SecureDet’s superior capability in handling extreme size variations. See Table A1 for detailed quantitative metrics. GT” denotes “Ground Truth”.

The inherent density and overlap in X-ray security imagery, exemplified by objects such as coiled cables and stacked batteries, are associated with formidable challenges for target detection. As shown in Figure 9, multi-scale detection performance analysis demonstrates SecureDet’s superior capability in handling extreme size variations. Ground Truth (GT) accurately annotate all critical targets; however, considerable deficiencies in existing methods have been observed in these demanding scenarios. Fast R-CNN is capable of identifying principal cable bundles but frequently fails to capture fine-grained details. EfficientDet is characterized by broad detection coverage but suffers from imprecise bounding box localization. Limited efficacy has been exhibited by RT-DETR in dense overlap regions, which impedes its capability to resolve complex, interwoven structures. Baseline models, including YOLOv8s, YOLOv10s, and YOLOv13s, have demonstrated compromised accuracy. In stark contrast, SecureDet-s achieves superior performance, with results being highly consistent with GT and the successful identification of all major contraband items. This advancement has been highlighted as a potential enhancement for security screening.

#### 4.5.5. Attention Visualization of Feature Learning and Feature Impact

**Figure 10:**
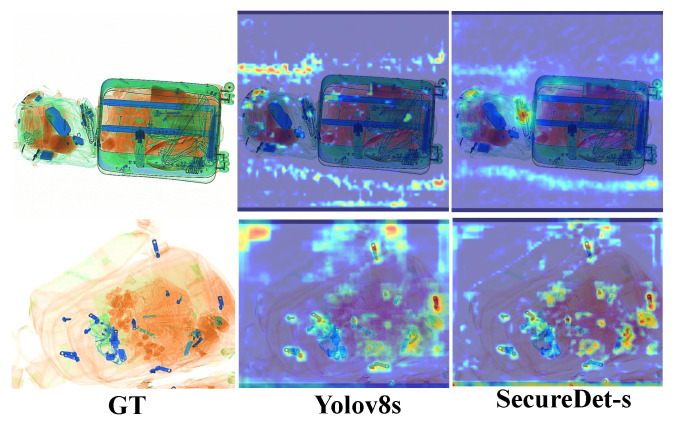
Grad-CAM-based attention visualization across different architectures (YOLOv8s and SecureDet-s) on representative X-ray scenarios The highlighted regions indicate salient features driving the final predictions.

To elucidate differences in feature learning across various architectures and their impact on detection performance, we employed Grad-CAM analysis. As shown in Figure 10, Grad-CAM-based attention visualization reveals the salient features driving detection decisions across different architectures. This technique generated attention maps focusing on key layers within each detector. Standardized procedures and normalized attention intensities facilitated consistent and equitable cross-model comparisons. Under conditions of severe occlusion, the baseline architecture (YOLOv8ss) demonstrated diffused, background-biased attention. Conversely, SecureDet-s directed its focus to intrinsic structural features of the target, such as edges, material boundaries, and connection points. It also effectively leveraged contextual information to pinpoint the unobscured portions of contraband.

## 5. Discussion

This study introduced SecureDet, a specialized YOLOv8-based architecture for X-ray contraband detection. Marked improvements in detection accuracy were demonstrated through our experimental results, particularly for occluded, small, and multi-scale objects, while real-time inference speeds were maintained. The findings are contextualized within the broader field of data-driven modeling, and the core scientific contributions of this work are discussed.

### 5.1. Architectural Synergy as the Core Contribution

A primary critique of application-specific deep learning models is that they often appear to be mere assemblages of pre-existing components. While the foundational concepts employed in SecureDet—such as channel-spatial attention, bidirectional feature fusion, and deformable alignment—are indeed established in the computer vision literature, our primary contribution is not the invention of these primitives. Rather, the novelty of this work lies in the discovery and validation of their non-trivial, synergistic integration tailored specifically for the challenges of X-ray contraband detection. This approach aligns with a growing trend in specialized manufacturing and inspection domains, where the intelligent combination and interpretation of machine learning models are paramount [46,47].

The most compelling evidence for this assertion is presented in the ablation study (Table 5). A critical architectural dependency was demonstrated: the isolated application of a weighted fusion neck (BiFPN) on the baseline YOLOv8 backbone was proven to be detrimental, resulting in a degradation of performance by 1.36% mAP@0.5. This finding is significant and non-obvious; it is shown that a theoretically powerful module may become counterproductive if it operates on features that are not adequately conditioned. This phenomenon, wherein advanced models fail in the absence of proper data or feature pre-processing, constitutes a recurring challenge in machine learning applications, ranging from medical imaging to industrial process control. A clear, domain-specific example of this principle has been provided by this work. The success of SecureDet is derived from a clear design principle validated by the results obtained.

- **Enhance First:** The RFCBAMConv module first enriches the backbone features, making them more discriminative and robust to noise and blur. This is a crucial step, as standard feature detectors often show degraded performance when images are blurred, which is a common issue in low-lighting or rapid-scan conditions [48].
- **Align Second:** The ECFA and ASFA modules then explicitly model contextual relationships and correct for spatial misalignments across feature hierarchies.
- **Fuse Last:** Only after features are enhanced and aligned does the BiFPN module effectively fuse multi-scale information, leading to the peak performance of the full SecureDet model.

The contribution of SecureDet is characterized as an architectural blueprint, which establishes a necessary sequence of operations for this problem domain. It has been demonstrated that, in the context of X-ray imagery, the order and interdependence of advanced components are more critical than their individual capabilities. This principle may inform the design of future detectors.

### 5.2. Mapping Architectural Innovations to X-Ray Image Characteristics and Remaining Challenges

A strong correspondence between specific architectural choices and the underlying physical characteristics of X-ray imaging has been revealed through a deeper analysis of the results. The success of SecureDet is attributed to a targeted approach designed to address the inherent challenges of the domain, which further assists in illuminating the model’s remaining limitations.

The ablation study presented in Table 5 demonstrates that the combination of ECFA and ASFA yielded the most substantial performance improvement, quantified as +1.50% mAP@0.5. As shown in Table A1, comprehensive statistical analysis provides detailed quantitative metrics across all evaluation scenarios, including precision, recall, and F1 scores for different detection challenges. This finding suggests that resolving contextual ambiguity and spatial misalignment constitutes a significant challenge in X-ray contraband detection. In X-ray images, object overlap is characterized not merely as occlusion but as a semi-transparent superposition, where pixel intensities are determined by the material densities and thicknesses of multiple objects along the beam’s path.

The Enhanced Contextual Feature Alignment (ECFA) module is designed to address the issue through the implementation of self-attention for the modeling of long-range dependencies. Through this methodology, inferences regarding the presence of an object, such as a firearm, can be made despite local features being compromised by overlapping items, as the visible components are related to a comprehensive understanding of the baggage content [49].

The importance of the Adaptive Spatial Feature Alignment (ASFA) module employing deformable convolution has been established. A capability to adjust the sampling grid for the purpose of concentrating on the feature points of the target object while disregarding the extraneous signals from occluding foreground or background items is provided. This methodology is classified as a data-driven approach to achieve object separation within a superimposed signal.

The challenge of separating superimposed objects from a 2D projection shares conceptual similarities with optimal transport problems, where one seeks to find an optimal coupling between different probability distributions [50]. RFCBAMConv alone yields a modest gain of +0.56% mAP@0.5, indicating limited standalone impact. The issue of extreme scale variation and blurred boundaries is addressed through the creation of a multi-scale feature representation prior to the application of attention [51]. This approach guarantees that the subsequent alignment and fusion modules are supplied with features that are inherently robust to the low-resolution and scattering effects typically encountered in security scanners.

However, the limitations of the model are regarded as informative as its successes. In the most challenging occlusion scenario (Figure 7, third row), SecureDet-s, despite outperforming all other models, registered one missed detection. This observation indicates a fundamental boundary of what purely data-driven, 2D models can achieve. When an object is heavily occluded to the extent that its distinctive features are almost entirely obscured by those of another object, a lack of sufficient information occurs, preventing confident detection. It is suggested that a powerful, implicit understanding of X-ray physics has been acquired by the model; however, failures emerge when the 2D projection becomes mathematically non-unique or ambiguous. This limitation serves to underscore the potential value of physics-based models [52], which, while computationally intensive [53], could resolve such ambiguities effectively [54]. Future inquiries may investigate a hybrid approach that employs a data-driven model for real-time prediction, while high-uncertainty cases could be flagged for analysis by a more slowly operating, physics-informed algorithm [55], thereby integrating the strengths of both paradigms.

### 5.3. Overcoming Data Scarcity and the “Black Box” Problem

A significant hurdle in the development of robust deep learning models for security and manufacturing applications arises from the scarcity of large, comprehensively annotated datasets. This challenge has been extensively documented in fields including medical imaging [56] and CFRP drilling analysis [57]. The initial training phase employed a private dataset; however, strong performance on public benchmarks, such as OPIXray and HiXray (Table 6), indicates the generalization capability of the model. Such outcomes suggest that architectural enhancements confer a degree of robustness, aiding in the mitigation of dependency on extensive training sets. This finding has been corroborated in studies employing techniques such as virtual sample generation to address data scarcity.

The “black box” nature of deep learning models is implicitly addressed through the work presented herein. By systematically deconstructing the architecture and evaluating each component’s contribution via ablation studies, a degree of interpretability is provided. The success of the ECFA and ASFA modules is linked to the physical challenges associated with object occlusion and spatial distortion in X-ray imaging. This validation of components serves as a practical, qualitative form of model inspection. Integration of more formal eXplainable AI (XAI) methods, such as Gradient-weighted Class Activation Mapping (Grad-CAM), is suggested for future work to visualize the model’s focus and further diagnose its decision-making process, akin to methodologies applied in electrochemical machining and acoustic monitoring [58,59]. Graph-based machine learning models [60] can also be used to facilitate the identification of relationships between detected items, with unusual co-occurrences potentially flagged as candidates for further evaluation. Such methods would enhance trust in the system and offer insights into potential failure modes, thereby improving system reliability for critical security deployment [61].

### 5.4. Implications and Future Directions

The performance of SecureDet is characterized by a robust and efficient framework for the enhancement of contraband detection in practical security scenarios. The accurate localization of heavily occluded and small items is recognized as a direct contribution to public safety, possibly resulting in a reduction of cognitive load on human operators and a minimization of false alarm rates. Understanding user willingness to adopt new security technologies is key, similar to how researchers study consumer preferences and barriers to adopting new mobility services [62]. Several future research avenues are identified based on this work:

- **XAI Integration:** Applying methods like SHAP and Grad-CAM to understand which input features (e.g., textures, material densities) the model prioritizes for different contraband types.
- **Semi-Supervised Learning:** Exploring non-fully supervised learning paradigms to leverage the vast amounts of unlabeled X-ray data available, thereby reducing the reliance on costly manual annotation and addressing the “label scarcity” challenge head-on.
- **Multi-Modal Fusion:** Incorporating data from other sensor modalities, such as dual-energy X-ray or 3D computed tomography (CT) scans, to provide richer information for detection.
- **Deployment and Optimization:** Further optimizing the model for deployment on edge devices with limited computational resources, ensuring its practical applicability in a wider range of security checkpoints.

## 6. Conclusions

This paper presents SecureDet, a novel YOLO-based framework that establishes new performance benchmarks for X-ray contraband detection by addressing fundamental limitations through principled architectural innovations. Synergistic components are integrated in the approach, including adaptive multi-scale feature extraction, uncertainty-aware fusion, cross-scale attention, and deformable convolution-based aggregation, which contribute to substantial performance gains (e.g., +2.97% mAP over YOLOv8s baseline) while real-time inference is maintained. Methodologically, the theoretical understanding of feature representation for challenging imaging conditions is advanced by SecureDet. Practically, enhancements are observed in security screening effectiveness and passenger experience, with broad potential for wider applications in fields such as medical imaging. A robust foundation for next-generation automated detection systems is provided by this systematic framework, along with clear future research directions.

### Limitations Under Extreme Occlusion and Future Directions

While SecureDet demonstrates robust performance under noise and clutter, extreme occlusion fundamentally limits its effectiveness. This limitation arises because severe occlusion removes critical discriminative evidence. Consequently, cross-scale fusion mechanisms may incorrectly weigh occluder textures, and spatial alignment strategies can lead to mis-sampling of occluded regions. Further compounding these issues, Non-Maximum Suppression (NMS) can suppress partially visible true positives when overshadowed by high-scoring false positives from clutter. Additionally, our training dataset contains insufficient examples with very high occlusion ratios.

To address these multifaceted challenges, our future research will focus on several key areas. We will implement advanced occlusion-aware data augmentation strategies, including techniques like Cutout, Hide-and-Seek, Random Erasing, and instance-level copy-pasting with realistic occluders. Architecturally, we aim to introduce a visibility-aware auxiliary head designed to judiciously gate feature fusion and re-calibrate detection confidences. Enhancements to deformable or iterative alignment mechanisms within ECFA/ASFA are also planned. Furthermore, we will explore adaptive post-processing techniques, such as Soft-NMS or class-aware NMS, to better handle competing detections. Concurrently, we intend to bolster context aggregation capabilities by leveraging larger receptive fields or more efficient lightweight attention mechanisms. To rigorously evaluate our progress in this challenging domain, we commit to reporting stratified recall and detailed error analyses categorized by occlusion level, complemented by targeted failure-case visualizations and ablation studies.

## Figures and Tables

**Table 1:** RFCBAMConv component hyperparameters (design-formula view).

Component	Setting
Channel attention bottleneck	two FC layers, reduction ratio r=16, activation: sigmoid
Spatial attention	$\left[\max_{c}\left(F_{\mathrm{unfold}}\right),\;{\mathrm{avg}}_{c}\left(F_{\mathrm{unfold}}\right)\right]\to {\mathrm{Conv}}_{3\times 3}\to$ sigmoid
Context construction	depth-wise separable ${\mathrm{Conv}}_{k\times k}$; spatial unfold (patch/stride: design-formula)
Attention application	sequential weighting: $M_{\mathrm{ch}}$ then $M_{\mathrm{sp}}$
Output layer	${\mathrm{Conv}}_{k\times k}$ with stride s=k; BatchNorm + ReLU

**Table 2:** Architectural scaling parameters of SecureDet variants.

Variant	Backbone	Depth Multiplier	Width Multiplier	Design Objective
SecureDet-n	YOLOv8n	0.33	0.25	Maximize efficiency for edge devices
SecureDet-s	YOLOv8s	0.33	0.50	Balance speed and accuracy for general scenarios
SecureDet-m	YOLOv8m	0.67	0.75	Pursue highest accuracy for high-performance servers

**Table 3:** Baseline training and inference configuration (compact).

Item	Setting
Input resolution	640×640
Batch size	16
Epochs	300
Optimizer	SGD (initial learning rate 0.01)
Losses	BCE (cls, obj) + CIoU (box)
Schedule	cosine with warmup; EMA; weight decay; light label smoothing (design-formula)
Augmentation	strong data augmentation (design-formula)
Early stopping	by validation mAP@0.5:0.95
Inference thresholds	confidence 0.25, NMS IoU 0.7
Metrics	Precision, Recall, mAP@0.5, mAP@0.5:0.95; Params, GFLOPs, FPS

**Table 4:** Performance comparison of different YOLO models.

Method	P (%)	R (%)	mAP@0.5 (%)	mAP@0.5:0.95 (%)	Params (M)	GFLOPs	FPS
YOLOv8s	91.065	72.725	79.287	66.379	13.8	28.6	83.4
YOLOv8m	83.59	81.3	81.32	68.3	25.9	78.9	45.3
YOLOv8n	86.894	64.541	73.339	56.079	3.2	8.8	122.3
Fast R-CNN	84.781	62.537	70.849	54.065	N/A	N/A	N/A
RT-DETR-R50	87.757	65.618	74.346	61.428	22.8	139.8	58.7
EfficientDet-d2	89.274	70.922	79.086	63.398	8.1	11.0	41.57
YOLOv10l	91.242	71.123	77.723	64.497	24.4	120.3	46.4
YOLOv10x	91.988	73.633	78.466	69.487	29.5	160.4	35.5
YOLOv11n	83.235	59.895	69.843	52.575	2.9	6.5	97.8
YOLOv11s	90.775	72.218	79.597	66.088	9.4	21.5	61.1
YOLOv11m	93.929	73.03	82.088	71.686	20.1	68.0	25.46
YOLOv13n	84.2	40.7	50.5	37.5	3.1	7.5	126.9
YOLOv13s	84.3	53.3	62.2	47.0	9.3	21.4	92.3
YOLOv13m	85.107	60.112	68.311	53.279	20.2	67.5	53.5
SecureDet-n	88.006	67.618	75.529	59.97	3.0	7.1	113.1
SecureDet-s	93.558	73.034	81.628	69.544	7.55	21.4	78.5
SecureDet-m	**92.602**	**74.611**	**82.258**	**72.312**	19.2	58.9	59.2

**Note:** Bold values indicate the best performance results among all compared methods for each metric.

**Table 5:** Ablation study results. SecureDet-s achieves its parsimonious 7.55 M architecture by replacing YOLOv8s’s substantial 11.13 M PANet neck with a lean BiFPN. Conversely, RFC+ECFA+ASFA augments the original neck, accumulating significantly more parameters (13.22 M).

Configuration	RFCBAM	BiFPN	ECFA	ASFA	P (%)	R (%)	mAP@0.5	mAP@0.5:0.95	Params (M)
YOLOv8s (Baseline)					91.07	72.73	79.29	66.38	11.13
only-BiFPN		✓			87.87	70.08	77.93	59.99	7.37
RFCBAM	✓				90.01	71.76	79.85	64.91	11.41
RFC+BiFPN	✓	✓			90.05	72.18	78.95	64.61	10.95
ECFA+ASFA			✓	✓	91.59	73.52	80.79	65.90	12.50
ECFA+ASFA+BiFPN		✓	✓	✓	89.88	69.11	77.88	61.75	7.07
RFC+ECFA+ASFA	✓		✓	✓	88.88	72.63	78.82	64.43	13.22
SecureDet-s (Full Model)	✓	✓	✓	✓	**93.56**	**73.03**	**81.63**	**69.54**	**7.55**

**Note:** ✓ indicates that the corresponding component/module is included in that configuration. Bold values indicate the best performance results achieved by our full SecureDet-s model compared to all ablation configurations.

**Table 6:** Performance comparison on different datasets.

Datasets	Methods	P (%)	R (%)	mAP@0.5 (%)	mAP@0.5:0.95 (%)
OPIXray	Fast R-CNN	81.065	72.725	79.287	38.379
	YOLOv8s	**90.466**	86.531	89.889	43.242
	RT-DETR	89.5	86.535	90.491	**54.469**
	EfficientDet	86.409	82.528	87.925	42.674
	YOLOv12s	84.781	62.537	89.849	42.065
	SecureDet-s	89.808	**87.106**	**91.156**	54.436
HiXray	Fast R-CNN	82.176	61.172	69.42	51.885
	YOLOv8s	87.832	70.47	78.099	62.295
	RT-DETR	**93.431**	73.481	81.259	68.345
	EfficientDet	90.18	76.73	80.982	50.011
	YOLOv12s	89.274	70.922	79.086	63.398
	SecureDet-s	84.795	**81.11**	**83.585**	**69.12**

**Note:** Bold values indicate the best performance results among all compared methods for each metric in the respective dataset.

**Figure A1:**
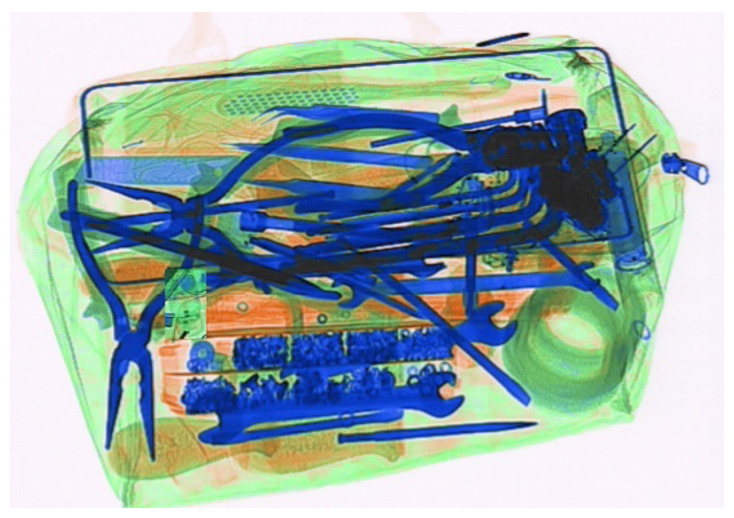
Original retrieved image corresponding to Figure 6 and Figure 9.

**Figure A2:**
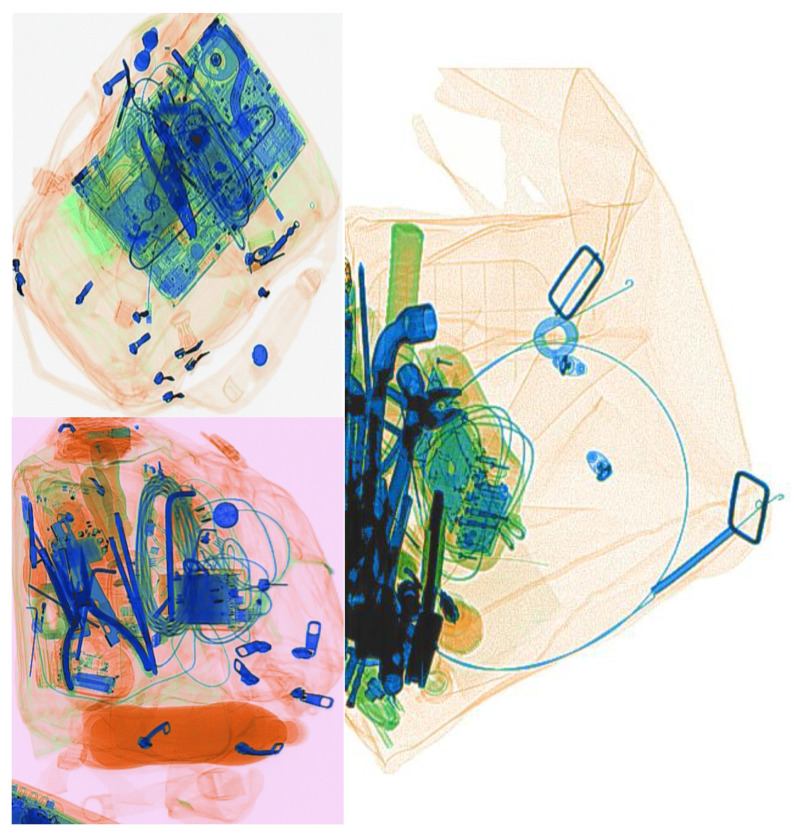
Original retrieved image corresponding to Figure 7 (severe occlusion scenario).

**Figure A3:**
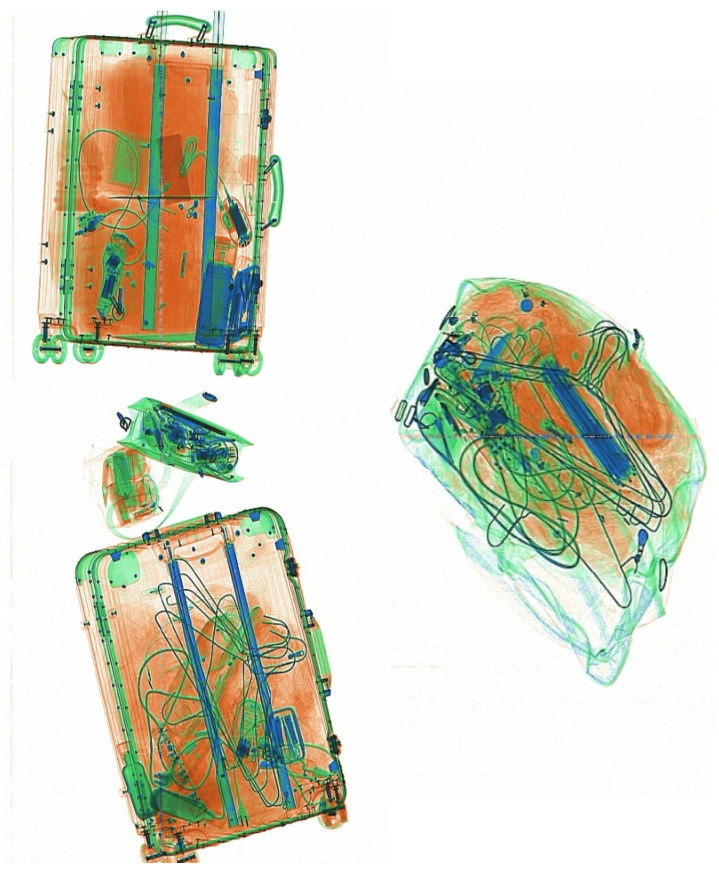
Original retrieved image corresponding to Figure 8 (small-object detection).

## Data Availability

The datasets used in this study are publicly available: OPIXray dataset can be accessed at https://github.com/OPIXray-author/OPIXray, accessed on 15 December 2024 and HiXray dataset can be accessed at https://github.com/HiXray-author/HiXray, accessed on 15 December 2024. The source code and trained models will be made available upon reasonable request to the corresponding author.

## Abbreviations

The following abbreviations are used in this manuscript:

YOLO	You Only Look Once
mAP	mean Average Precision
RFCBAMConv	Receptive Field CBAM Convolution
BiFPN	Bidirectional Feature Pyramid Network
ECFA	Enhanced Contextual Feature Alignment
ASFA	Adaptive Spatial Feature Alignment
CBAM	Convolutional Block Attention Module
FPN	Feature Pyramid Network
SGD	Stochastic Gradient Descent
GPU	Graphics Processing Unit

## Appendix A. Supplementary Statistical Analysis

**Table A1:** Comprehensive statistical analysis of detection performance for Figure 5, Figure 6, Figure 7 and Figure 8 across different methods.

Figure	Method	GT	TP	FN	FP	Precision	Recall	F1
Figure 6	only-BiFPN	3	1	2	0	1.000	0.333	0.500
	yolov8s	3	1	2	0	1.000	0.333	0.500
	RFCBAM	3	2	1	1	0.667	0.667	0.667
	RFC + BiFPN	3	0	3	2	0.000	0.000	0.000
	ECFA + ASFA	3	2	1	1	0.667	0.667	0.667
	ECFA + ASFA + BiFPN	3	2	1	1	0.667	0.667	0.667
	RFC + ECFA + ASFA	3	2	1	0	1.000	0.667	0.800
	SecureDet-s	3	3	0	0	1.000	1.000	1.000
Figure 7	Fast R-CNN	5	2	4	1	0.667	0.333	0.444
	EfficientDet	5	3	4	0	1.000	0.429	0.600
	RT-DETR	5	2	3	0	1.000	0.400	0.571
	YOLOv8s	5	2	3	0	1.000	0.400	0.571
	YOLOv10s	5	2	3	1	0.667	0.400	0.500
	YOLOv13s	5	2	3	0	1.000	0.400	0.571
	SecureDet-s	5	5	0	1	0.833	1.000	0.909
Figure 8	Fast R-CNN	5	2	3	1	0.667	0.400	0.500
	EfficientDet	5	2	3	1	0.667	0.400	0.500
	RT-DETR	5	2	3	0	1.000	0.400	0.571
	YOLOv8s	5	2	3	0	1.000	0.400	0.571
	YOLOv10s	5	2	3	0	1.000	0.400	0.571
	YOLOv13s	5	1	4	0	1.000	0.200	0.333
	SecureDet-s	5	5	0	0	1.000	1.000	1.000
Figure 9	Fast R-CNN	3	1	2	0	1.000	0.333	0.500
	EfficientDet	3	2	1	0	1.000	0.667	0.800
	RT-DETR	3	0	3	0	0.000	0.000	0.000
	YOLOv8s	3	2	1	0	1.000	0.667	0.800
	YOLOv10s	3	2	1	0	1.000	0.667	0.800
	YOLOv13s	3	2	1	0	1.000	0.667	0.800
	SecureDet-s	3	3	0	0	1.000	1.000	1.000

**Note:** GT = Ground Truth objects; TP = True Positives; FN = False Negatives; FP = False Positives; Precision = TP/(TP + FP); Recall = TP/(TP+FN); F1 = 2 × (Precision × Recall)/(Precision + Recall).

## Appendix B. Supplementary Retrieved Images for Zoom-In Comparison

To facilitate better visual comparison as requested for Figure 5, Figure 6, Figure 7 and Figure 8, we provide the original retrieved images in high resolution below; readers can directly zoom into the regions of interest to inspect fine details and occlusion boundaries.

These originals are provided to enable precise zoom-in comparisons of the cropped areas shown in the main figures.
